# A rare gas-containing lumbar synovial cyst treated by percutaneous transforaminal endoscopic cystectomy: A case report and literature review

**DOI:** 10.3389/fsurg.2023.1095572

**Published:** 2023-03-21

**Authors:** Ziran Wei, Qian Lin, Hao Zhang, Haoyun Zhang, Xuexiao Ma, Chao Wang

**Affiliations:** ^1^ Department of Sports Medicine, The Affiliated Hospital of Qingdao University, Qingdao, China; ^2^ Department of Spine Surgery, The Affiliated Hospital of Qingdao University, Qingdao, China

**Keywords:** synovial cyst, vacuum cleft, gas, endoscopic surgery, cystectomy

## Abstract

Spinal synovial cysts are rare entities for which standard surgical strategies are inconsistent. Here, we present an uncommon intraspinal gas-containing synovial cyst treated by percutaneous transforaminal endoscopic cystectomy. A 52-year-old man presented with radicular pain and intermittent claudication that had persisted for one month. Computed tomography revealed an intraspinal cystic lesion anteromedial to the left L4-L5 articular joint and the center of the lesion manifested gas contents. A transforaminal endoscopic procedure was performed and confirmed as a safe and minimally invasive technique for gas-containing lumbar synovial cysts. It provides a valuable substitution and supplementation to open surgery.

## 1 . Introduction

Spinal synovial cysts are uncommon lesions that predominantly occur within the lumbar spine. The underlying formative mechanisms of synovial cysts include spinal instability, degenerative spondylolisthesis, and facet joint arthritis (1). Most synovial cysts are asymptomatic and, hence, are incidentally found on magnetic resonance imaging (MRI). Intraspinal synovial cysts, however, may exhibit symptoms such as myelopathy and radiculopathy. Surgical resection with or without fusion and instrumentation is considered for patients who do not respond to ongoing conservative therapy. Generally, postoperative relief is prompt and sustainable; however, recurrence has been noted (2, 3).

Synovial cysts containing gas are even rarer in the clinic. To our knowledge, only 9 cases of gas-containing lumbar synovial cysts have been reported in the English literature thus far, most of which have been treated by open cystectomy (4–12). Here, we present a novel case of gas-containing lumbar synovial cysts treated percutaneously using transforaminal endoscopic technique. Further, we have reviewed the literature to provide a comprehensive understanding of this uncommon entity.

## 2 . Case presentation

A 52-year-old man referred to our department for radicular pain and intermittent claudication that had persisted for one month. The onset of the pain was insidious, and the patient denied a history of trauma. The pain radiated from the left hip to the posterolateral aspect of the left leg and ankle. The pain was exacerbated by walking, whereas it could be immediately relieved by bed rest or sitting down. The patient tried anti-inflammatory medications but the effect was unsatisfying.

**Figure 1:**
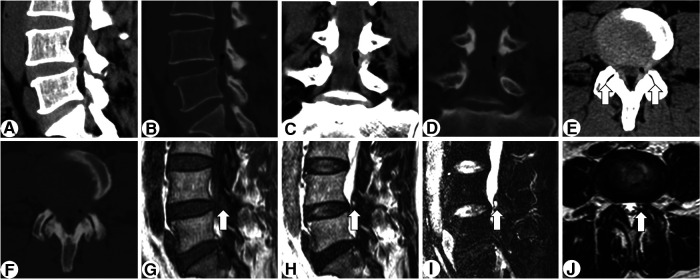
Preoperative CT and MRI of the lesion. Sagittal, coronal, and axial CT scan (**A-F**) revealing a gas-containing intraspinal cystic lesion anteromedial to the left L4/5 articular joint and bulging to the lateral canal. Osteoarthritis and vacuum clefts (arrow) are observed at the bilateral L4/5 facet joints. MRI (G-I) revealed a hypointense lesion on T1-weighted image, but a hyperintense mass with a hypointense rim (arrow) on both T2-weighted and fat suppression images.

On physical examination, flexion, extension, and lateral bending of the lumbar spine were not restricted. Mild L4-L5 interspinous tenderness was detected, and hypoesthesia was present over the left L5 nerve dermatome. Full muscle strength was recorded for the lower extremities, and both knee and ankle reflexes were symmetrical and active. Straight leg raising tests revealed negative results. Computed tomography (CT) found an intraspinal cystic lesion adjacent to the ligamentum flavum, anteromedial to the left L4-L5 articular joint, bulging to the lateral aspect of the canal, and leading to lateral recess stenosis (Figures 1A–F). Remarkably, the center of the lesion manifested a round shape (axial plane) or tadpole shapes (sagittal and coronal planes) with void density, which indicated gas contents. Osteoarthritis and vacuum cleft phenomenon was found at the bilateral L4-L5 facet joints. Nevertheless, the gas in the lesion was seemingly discontinued with the vacuum cleft in the facet joint. MRI revealed a hypointense lesion on T1-weighted image, but a hyperintense mass with a hypointense rim on both T2-weighted and fat suppression images (Figures 1G–J). The intervertebral disc showed minimal degenerative change, and no vacuum phenomenon was discovered. Based on the clinical and image findings, a primary diagnosis of synovial cyst was made.

**Figure 2:**
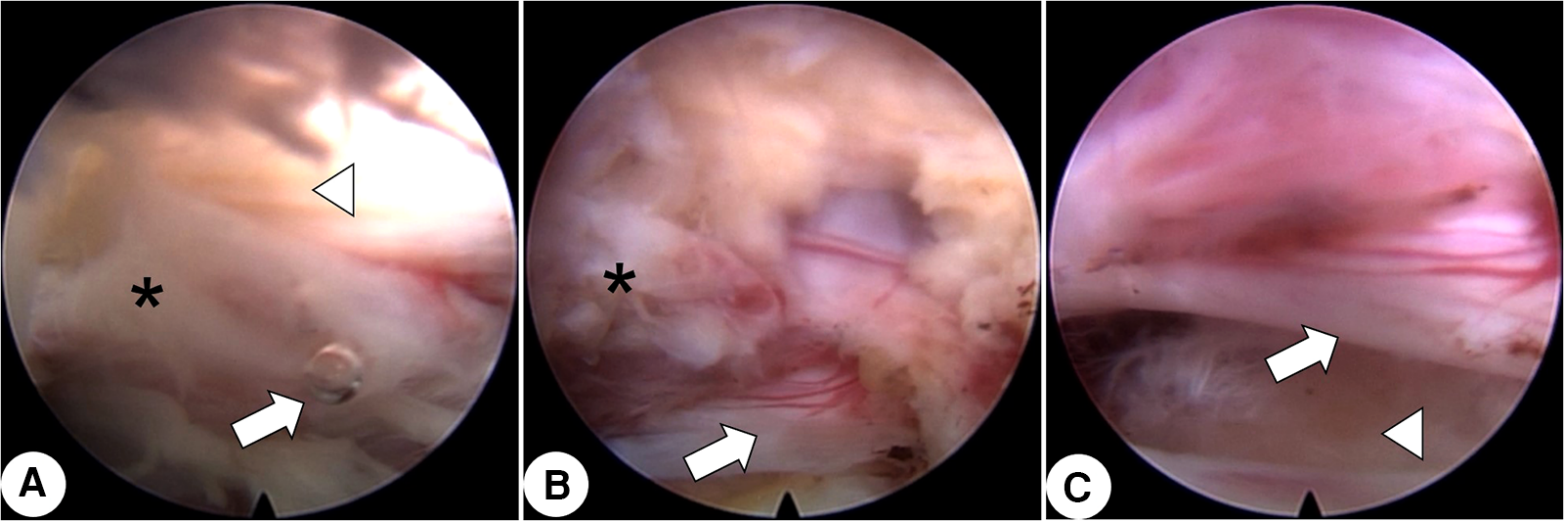
Intraoperative endoscopic images. (**A**) After facetectomy with a trephine, the cyst (asterisk) with a fistula connecting to the resected facet joint is seen. An extruded bubble (arrow) indicates gas in the cyst. The medial ligamentum flavum is shown (arrowhead). (**B**) After gradual piecemeal resection of the cyst (asterisk), the impinged L5 nerve root (arrow) is seen. (**C**) The space beneath the nerve root (arrow) is shown and the annulus fibrosus (arrowhead) is shown to be intact.

**Figure 3:**
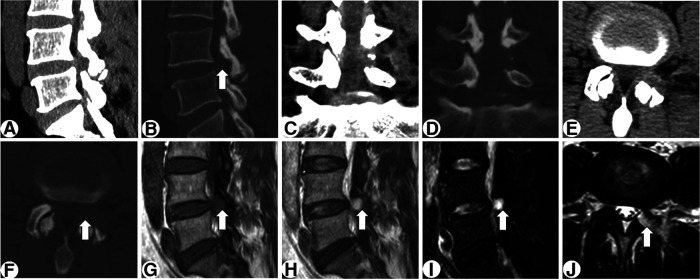
Postoperative CT (**A-F**) and MRI (**G-J**) demonstrating a bony trajectory (arrow) targeting the lesion and complete decompression of the nerve root and dura sac.

A transforaminal endoscopic procedure with local anesthesia was planned. After partial facetectomy with an endoscopic trephine, a cyst with a fistula connecting to the resected facet joint appeared in the endoscope field (Figure 2A). Bubbles extruding from the cyst confirmed the target, and the cyst, together with the surrounding ligamentum flavum, was resected piecemeal. Subsequently, the impinged L5 nerve root gradually arose (Figure 2B). Following complete cystectomy and clearance of the ligamentum flavum, the space beneath the nerve root appeared, and the annulus fibrosus was found to be intact and therefore fully preserved (Figure 2C). The patient experienced complete pain relief after surgery and no complication occurred. After six months, the patient maintained no signs of recurrence. Postoperative CT and MRI demonstrated a bony trajectory targeting the lesion and full decompression of the nerve root and dura sac (Figure 3).

## 3 . Discussion

A more common intraspinal gas-containing lesion is the disc herniation or discal cyst, both of which are characterized by predominant disc degeneration and vacuum phenomenon of the disc. The lesion is usually connected to the annulus fibrosus and impinges on the dura sac or the nerve root from the ventral side. On the contrary, as showed in our patient, an intraspinal gas-containing synovial cyst generally manifests minimal degeneration of the disc but obvious arthritis and vacuum clefts of the facet joints. The neural structures commonly suffer from compression at the dorsolateral direction. Intraspinal synovial cyst should also be distinguished from neurogenic tumor, such as cystic schwannoma. An enhanced MRI is helpful in differentiation since the cystic schwannoma typically shows ring enhancement while no enhancement is seen for the synovial cyst. Moreover, the gas-containing feature strongly indicates a degenerative, rather than a neoplastic lesion. Thereafter, the patient was not required for an additional enhanced MRI preoperatively.

To our knowledge, only 9 cases of gas-containing spinal synovial cysts have been previously published in the English literature. The key characteristics of the previously published 9 cases and this present case are presented in Table 1. Most cases pertained to patients in their fifth to seventh decade of life and had a slight male predominance. The cysts tended to occur at L4-L5, the level of maximum mobility, which was speculated to be related to facet joint arthritis and segmental instability (1). A majority of the cases (8/10) showed an adjacent vacuum cleft of the facet joint. The formation of synovial cyst is postulated as a check valve mechanism, similar to that of gas-containing disc herniation (13, 14). A pathological study demonstrated that the synovial cyst communicates with the facet joint by a bursa-type channel within the ligamentum flavum (13). The progressive osteoarthritis of the facet joint releases bony and cartilaginous debris into the synovial fluid, which may escape into the channel and lodge in the wall where it induces granulation tissue formation. The pathological change further results in the check valve effect of the synovial cyst. Most gas-containing synovial cysts identified a concurrent vacuum cleft of the ipsilateral facet joint, which suggests that both the gas-containing and fluid-only contents share the similar formation mechanism. However, it is believed that the gas is unlikely to be spontaneously absorbed (11). This is evidenced by the fact that most of the patients enrolled in our review (Table 1) eventually underwent open or endoscopic surgeries.

**Table 1:** Literature review of spinal synovial cysts containing gas.

Case No.	Year & Authors	Age	Sex	Level	Major complaints	Non-surgical therapy	Facet joint vacuum cleft	Surgery
1	1983, Spencer et al.	47	F	L3/4	LBP and RP	N/A	Yes	Open cystectomy
2	1984, Schulz et al.	57	M	L4/5	LBP	N/A	Yes	Open cystectomy
3	1987, Beaty	73	M	L4/5	RP	N/A	No	Open cystectomy
4	1987, Fardon et al.	74	F	L4/5	LBP and RP	Medication	N/A	Open cystectomy
5	1992, Tobback et al.	70	M	L4/5	LBP and RP	N/A	Yes	N/A
6	2000, Firth	70	M	L4/5	RP	Chiropractic and medication	Yes	Open cystectomy
7	2013, El Beltagi et al.	51	F	L4/5	LBP and RP	Medication	Yes	No
8	2016, Cebeci et al.	55	F	L4/5	LBP and RP	N/A	Yes	N/A
9	2020, Sugishima et al.	68	M	L4/5	RP	Medication and nerve block	Yes	Open cystectomy
10	Current case	52	M	L4/5	RP	Medication	Yes	Endoscopic cystectomy

LBP, low back pain; RP, radicular pain.

Cystectomy, whether by open or endoscopic procedure, achieved satisfactory clinical results for patients resistant to conservative therapies. Percutaneous endoscopic technique provides a novel avenue for surgical intervention. A recent case series preliminarily elucidated the feasibility and effectiveness of this technique in the treatment of lumbar synovial cysts (15, 16). Compared to open surgery, endoscopic techniques show compelling advantages in terms of prompt recovery, minimal structural disturbance, and cost-effectiveness (16). Technically, either the transforaminal or translaminar approach can be chosen. For the resection of a L4-L5 lesion or above, we usually preferred a transforaminal approach considering the wide foramen space and small interlaminar window, especially for patients with local anesthesia. For the L5-S1 level, a translaminar approach is often the choice for the block of iliac crest on the trajectory of transforaminal approach, as well as the advantage of larger interlaminar window. Nevertheless, the approach should be decided individually according to various factors, such as surgeon's preference, type of anesthesia, imaging findings, and comorbidities (15).

In this case, we facilitated the cystectomy by the transforaminal approach and an additional facetectomy to directly target the lesion. Considering the close relationship between the cyst and the degenerative facet joint, limited removal of the adjacent ipsilateral facet joint and accessories may help reduce the risk of recurrence whilst preserving segmental stability. The water medium makes the gas-containing lesion more distinguishable and easier to identify since the gas in the cyst will be expelled when the cyst is cut open under monitoring. One of the limitations focuses on the concerns of segmental stability, which warrants long-term follow-up. Another limitation is that unlike open surgery, in which the specimen can be obtained *en bloc*, piecemeal resection of the cyst in the endoscopic surgery gets only tiny shreds, most of which is washed away by flushing saline. Thereafter, the sample for pathology was not deliberately collected. Emphasis shall be given for the importance of the definitive pathological diagnosis in future.

In conclusion, percutaneous endoscopic cystectomy is a safe and minimally invasive technique for gas-containing lumbar synovial cysts. It provides a valuable substitution and supplementation to open surgery. Long-term follow-up is warranted for evaluation of segmental stability and recurrence.

## Data Availability

The original contributions presented in the study are included in the article/Supplementary Material, further inquiries can be directed to the corresponding authors.
